# Advances in pediatrics in 2023: choices in allergy, analgesia, cardiology, endocrinology, gastroenterology, genetics, global health, hematology, infectious diseases, neonatology, neurology, pulmonology

**DOI:** 10.1186/s13052-024-01818-3

**Published:** 2024-11-14

**Authors:** Carlo Caffarelli, Francesca Santamaria, Elena Bozzola, Bertrand Tchana, Ettore Piro, Enrico Vito Buono, Daniela Cunico, Raffaele Cerchione, Alessandro Dorato, Cristina Fontanella, Sergio Bernasconi, Giovanni Corsello

**Affiliations:** 1 Clinica Pediatrica, Department of Medicine and Surgery, Azienda Ospedaliero Universitaria, University of Parma, Parma, Italy; 2 Department of Translational Medical Sciences, Federico II University, Naples, Italy; 3 Department of Pediatric, Pediatric Diseases Unit, IRCCS Bambino Gesù Children’s Hospital, Rome, Italy; 4 Cardiologia Pediatrica, Azienda Ospedaliero Universitaria, Parma, Italy; 5 Department of Sciences for Health Promotion and Mother and Child Care “G. D’Alessandro”, University of Palermo, Palermo, Italy; 6 Microbiome Research Hub, University of Parma, Parma, Italy

**Keywords:** Allergy, Analgesics, Cardiology, Endocrinology, Gastroenterology, Genetics, Infectious diseases, Neonatology, Neurology, Pulmonology

## Abstract

In the last year, there have been many remarkable articles published in the Italian Journal of Pediatrics. This review highlights papers that can be potentially helpful in healthcare practice among the most cited or accessed papers on the journal website. We have chosen key articles on allergy, analgesics, cardiology, endocrinology, gastroenterology, genetics, global health, infectious diseases, neonatology, neurology and pulmonology. Advances in understanding risk factors, mechanisms, diagnosis, treatment options and prevention of pediatric diseases have been discussed and in the context of the subsequent steps. We think that progresses achieved in 2023 will have a significant impact on the management of diseases in childhood.

## Background

The most important papers from distinct specialties that were published in the Italian Journal of Pediatrics in the first half of 2023 have been included in this review. We have selected key information based on those articles that were most cited or accessed on our website.

The aim is to provide an overview of the most influential published papers of the past year in the fields of allergy, analgesia, cardiology, endocrinology, gastroenterology, genetics, global health, infectious diseases, neonatology, neurology and pulmonology. The papers in our analysis covered a variety of novel insights in risk factors, mechanisms, diagnosis, treatment options and prevention. The advances that were more relevant in clinical practice, have been commented looking to the future.

## Allergy. 1- Eosinophilic gastrointestinal disorders; 2-  Mastocytosis

### Eosinophilic gastrointestinal disorders

Eosinophilic gastrointestinal disorders (EGID) are a group of disorders characterized by pathological eosinophilic infiltration of the esophagus, stomach, small intestine or colon, leading to organ dysfunction and clinical symptoms. In recent years there has been an increase in reports of Eosinophilic Esophagitis (EoE) above all [1]. Votto et al. [2] studied 60 patients with EGIDs. EGID diagnosis was made approximately 12 months after symptom onset, which was shorter than the delay observed in other studies [3]. However, the diagnosis was delayed in children with EoE who had failure to thrive and feeding problems than in children without growth and feeding problems. So, a prompt diagnosis is crucial to prevent failure to thrive. Furthermore, they observed an increased frequency of coexisting allergic diseases, especially food allergy. An elimination diet is beneficial in most children with EoE. These elements can indicate that a mixed IgE-mediated non IgE-mediated mechanism can be involved in the pathogenesis. An oral food challenge to the food in question [4] would be necessary to reach the diagnosis.

### Mastocytosis

Pediatric mastocytosis is a rare and heterogeneous group of disorders characterized by an abnormal clonal expansion of mast cells that accumulate in the skin (Cutaneous Mastocytosis) and/or, less frequently, in other organs or tissues (Systemic Mastocytosis). The release of mast cell mediators, including histamine and other vasoactive substances, is responsible for the clinical manifestations. Cutaneous Mastocytosis is defined by typical skin lesions with a positive Darier’s sign. Diagnosis of systemic mastocytosis is based on organ enlargements, elevated serum tryptase levels, cytoreduction and characteristic histopathological findings in biopsies of affected tissue [5]. Children with systemic mastocytosis are at risk of severe reactions due to mediator release mainly induced by allergens such as hymenoptera venom, foods [6], non IgE-mediated stimuli [7] or spontaneous. The management is based on identifying of triggers by IgE tests [8]. It is aimed at preventing the release of mast cell mediators and controlling symptoms with second-generation anti-H1 antihistamines, systemic corticosteroids and organ specific drugs. Bossi et al. [9] highlighted that a child affected by systemic mastocytosis with persistent rash, diarrhea, abdominal pain, palpitations, musculoskeletal symptoms, fatigue, refractory to anti-H1 and oral steroids became quickly asymptomatic following administration of omalizumab, a monoclonal antibody against IgE. Symptoms recurred when omalizumab was suspended. The child responded to restart of omalizumab. Side effects to omalizumab were not recorded.

## Analgesics. 1- Adverse reactions to ibuprofen or paracetamol; 2-  Pain in emergency department; 3-  Pain in surgery, oncology and hematology

### Adverse reactions to ibuprofen or paracetamol

Type 1 (or type “A”, Augmented) adverse reaction to drugs are dose-dependent, related to the pharmacologic mechanism and occur in normal subjects. Type 2 (or type “B”, Bizarre) are not dose-dependent, unrelated to the pharmacologic mechanism and occur in predisposed subjects. They include anaphylaxis and severe cutaneous reactions [10, 11]. In children, first-line treatment for mild-to-moderate pain and fever is either ibuprofen or paracetamol that have similar safety and tolerability profiles [12]. Marano et al. [13] analyzed 351 patients who contacted the hospital’s pediatric poison control center (PPCC) for exposure to ibuprofen and paracetamol from January 1, 2018 to September 30, 2022, to assess the incidence of any adverse reactions. Misuse or accidental ingestion was the most common reason for inappropriate oral use of paracetamol or ibuprofen, with a fifth of patients taking it for suicidal purposes. Most patients were not intoxicated and hospitalization was necessary for 30.5% of children. Type 1 adverse reactions were recorded in 10.8% of patients taking paracetamol and in 10.1% of cases after ibuprofen. The most common adverse reactions to paracetamol were vomiting, hypertransaminasemia, coagulopathy and headache those to ibuprofen were nausea, vomiting, abdominal pain, increased serum creatinine and dizziness.

### Pain in emergency department

Pain is one of the most frequent reasons for referral to pediatric emergency department, especially in younger children and those with special needs, a category in which undertreatment of pain (so-called “oligoanalgesia”) is very common. Oligoanalgesia is related to long-term negative behavioral and psychological consequences. Management of pain and anxiety is of fundamental importance and good pain control could help the entire medical team in the evaluation and treatment of a child [14]. Several studies have shown that very often the treatment of pain in children is inadequate and have highlighted the importance of adequate pain treatment in terms of immediate but also future well-being and neurological development of the patient [15, 16]. Bevacqua et al. [17] report the current state of the art of pediatric sedation and analgesia in Italian emergency rooms and identify existing gaps that need to be addressed. The survey proposed a case vignette and questions addressing different domains, such as pain management, availability of medications, protocols and safety aspects, staff training, and availability of human resources regarding sedation and procedural analgesia. Eighteen Italian sites participated in the study, 66% of which were represented by University Hospitals and/or Tertiary Care Centres. It was found that 27% of patients receive inadequate sedation. In many emergency departments there is a lack of availability of some drugs such as nitrous oxide, lack of use of intranasal fentanyl and topical anesthetics at triage, the use of safety protocols and pre-procedural checklists is rare, there is also a lack of staff training and lack of space. Moreover, the availability of child life specialists and hypnosis, as a non-pharmacological practice of sedation and analgesia is insufficient. The study highlights that, although much progress has been made in recent years in the treatment of pain in the pediatric emergency department setting, there is still much work to be done due to the complexity of pediatric patients and, sometimes, the need of adequate instruments/medicines as well as training of health personnel.

### Pain in surgery, oncology and hematology

Pain control is universally recognized as a human right and the correct assessment of pain is now one of the standards for the accreditation of health institutions. Proper pain management can reduce the incidence of complications, reduce hospital stays, achieve faster discharges and decrease the use of hospital resources. On the contrary, inadequate pain management can lead to persistent or chronic pain, alterations in nociception and emotional and psychological complications; pain can have negative effects on the physical and mental conditions of hospitalized patients, worsening the quality of life and increasing costs [18].

However, the assessment and especially the treatment of pain are still important health problems in hospitalized patients [19]. Marchetti et al. [20] compare a one-day survey that analyzed the prevalence of pain, pain intensity and pain therapy conducted in 2016, in which they showed suboptimal pain management in the surgery and oncohematology departments, with the same survey conducted in 2020. They found a higher prevalence of moderate/severe pain in the 2020 survey compared to the previous 2016 survey, both during hospitalization and in the 24 h preceding the day of the survey, despite hospital training initiatives aimed at doctors and nurses on pain therapy. On the other hand, the daily prescription of pain therapy has significantly improved both in terms of time indications and as needed. There were fewer children who were not prescribed any pain therapy compared to the 2016 survey. However, the quality of analgesic therapy was low in 2020 also compared to 2016. Indeed, the therapy administered led to a statistically significant undertreatment of pain, and it was unable to alleviate moderate/severe pain. Basically, many steps forward still need to be made, not so much in the evaluation, which most of the time appears correct, but in the correct use of drugs, also in relation to the type of pain [21].

## Cardiology. 1- Intravenous immunoglobulin in Kawasaki disease; 2- Acute myocarditis; 3- Prevention of respiratory syncytial virus infection in infants with congenital heart disease

### Intravenous immunoglobulin in Kawasaki disease

Kawasaki disease (KD), although a typically, a self-limited condition, lasting for an average of 12 days without therapy, is the main cause of acquired heart disease in western countries, as far as patients may develop cardiovascular complications, mainly coronary arteries aneurism, with life threatening issues such as coronary occlusion and sudden cardiac death. Treatment with intravenous immune globulin (IVIG) have dramatically change the outcome of KD, because of effectiveness in preventing coronary arteries abnormalities, and decreasing frequency of coronary arteries aneurysm development. Timely diagnosis and treatment are critical for the clinical outcome, but although a general consensus on immunoglobulin as first line treatment, optimal timing, with or without adjunctive therapy, is still debated [22, 23], with immune globulin resistance as a matter of concern [24] because of correlation with earlier therapy (within 4 days of disease) and coronary arteries aneurism development, as shown in a large review and meta-analysis. Several studies aimed to determine the optimal window for IVIG therapy, and although some controversies, starting within 7 days of illness seems to be the best [25].

### Acute myocarditis

Pediatric myocarditis is a challenging inflammatory disease because of the wide spectrum of clinical signs and symptoms, the multiple etiologies, and the complications and sequelae ranging from hemodynamic instability, ventricular dysfunction, dilated-cardiomyopathy, life-threatening arrhythmias and sudden cardiac death. Though improvement in the understanding of pathogenesis, several studies and attempts at meta-analysis, optimal treatment remains controversial and debated, because of small sample sizes and the quality of studies [26]. In addition to standard supportive care for heart failure and arrhythmias, current therapeutic strategies look for etiologically oriented treatment [27]. Anti-inflammatory and immune responses modulating agents have been considered beneficial [26, 28], in particular corticosteroids and IVIG for their broad and overlapping effects. If no treatment has demonstrated significant improvement in reducing the risk of mortality, corticosteroids seem to produce significant effects on left ventricle ejection, as shown in a meta-analysis [28], even if treatment effects are difficult to ascertain as far as ventricular function improves fully in many patients [26, 29].

### Prevention of respiratory syncytial virus infection in infants with congenital heart disease

Respiratory syncytial virus bronchiolitis is the leading cause of hospitalizations for infants and children under 2 years of age. Patients with hemodynamically significant congenital heart disease have a higher rate of hospitalization, need for intensive care and ventilator support [30, 31]. Full passive immune prophylaxis with palivizumab prophylaxis has shown to be effective against respiratory syncytial virus (RSV) infections, thus reducing RSV-related hospitalization rate, morbidity, and mortality, avoiding delay in interventional and surgical procedures in this category of patients [31, 32]. Although cost-effectiveness is still debated, it may impact healthcare resource availability and utilization [33].

## Endocrinology. 1- Diabetic ketoacidosis; 2-  Vitamin D; 3-  Treatment and prevention of obesity

### Diabetic ketoacidosis

Rates of diabetic ketoacidosis (DKA) at diagnosis vary from 11 to 80% depending upon region, even in developed countries. The risk of DKA in children after diagnosis of type 1 diabetes mellitus (T1D) is 1–10/100 person-years. DKA is usually provoked by intentional or inadvertent insulin omission, sometimes associated with intercurrent illness and increased insulin requirement [34]. It has been shown that more than 50% of diabetic children were treated in the pediatric intensive care unit (PICU) due to DKA in Croatia [35]. Passanisi et al. [36] showed that 51.5% of 103 children and adolescents with a new diagnosis of T1D had DKA and 10 subjects with T1D onset needed to be treated in PICU for severe clinical manifestations. Among these four children were younger than 5 years of age. Acute kidney injury was the most common complication of DKA followed by cerebral oedema, papilledema and acute esophageal necrosis. The authors emphasized that increased public awareness campaigns should be promoted to facilitate the recognition of early symptoms of diabetes and to reduce the morbidity and mortality associated with DKA.

### Vitamin D

Galeazzi et al. [37] report that in children aged between 5 and 10 years, living in a coastal area of Central Italy (Ancona) and subjected to screening for celiac disease, blood values of 25-hydroxyvitamin D (25(OH)D were sufficient in 36% of subjects, according to the classification proposed by a recent Italian Consensus which considers such values to be ≥ 30 ng/ml (≥ 75nmol/L). 21% had values classifiable as deficient (10–20 ng/ml) and 6% as severely deficient (< 10 ng/ml). It should be remembered that, in general, values < 12 ng/ml are considered at risk of rickets as confirmed by an extensive review on children aged under 4 years with radiological signs of rickets from which it appears that over 60% have values below this limit [38]. The prevalence data found in the study is substantially in line with epidemiological studies carried out in various European countries regardless of latitude. This confirms that other factors, in addition to sun exposure, which differs in the various latitudes, can play a significant role. In particular, socioeconomic conditions, lifestyles and eating habits must be taken into account. Furthermore, the study reports a higher percentage of deficient values in subjects of non-Caucasian ethnicity and in obese subjects. The latter have been and are the subject of various studies aimed at explaining the reason for this phenomenon [39]. The causes are not yet fully clarified and it is thought that various factors may contribute, such as: less sun exposure of this group due to decreased outdoor activities; vitamin D sequestration in the adipose tissue or uptake by this tissue; impaired liver vitamin D synthesis in fatty liver of severely obese subjects. The high prevalence of children with vitamin D deficient levels is stimulating a broad discussion on the modalities of a prophylaxis that is today recommended with a defined empirical modality due to the absence of strong scientific evidence but which, ideally, should be personalized at the individual level or at risk subpopulations, taking into account the specific individual needs and the type of pathology, especially extra-skeletal, that one wants to prevent [40].

### Treatment and prevention of obesity

Obesity is today considered, due to its prevalence, which continues to increase in various populations, and to the known risk of complications, such as cardiometabolic, psychosocial comorbidity and premature mortality [41]. a chronic disease of primary interest for public health. In 2023, a joint task force of the Italian Society of Pediatric Endocrinology and Diabetology, the Italian Society of Pediatrics and the Italian Society of Pediatric Surgery developed a consensus position statement on the treatment of obesity in children and adolescents [42]. Lifestyle intervention is the first step in treatment. In children over the age of 12, pharmacotherapy is the second step and bariatric surgery is the third, in selected cases. There are new developments in the medical treatment of obesity [43]. In particular, new medicines have demonstrated efficacy and safety and have been approved for use in adolescents [44]. The Food and Drug Administration has approved once-daily liraglutide, orlistat, and phentermine–topiramate for adolescents at least 12 years of age; only liraglutide is approved by the European Medicines Agency.

On the other hand, origin is polyfactorial and the various ways of dealing with it at a therapeutic level have proven, especially in the long term, not particularly effective also due to the various barriers that can oppose it, making therapeutic success more unlikely [45]. For a long time, therefore, attempts have been made to develop prevention with a particular focus on pre-school age and the first school cycles. A recent review that only takes into consideration randomized controlled trials (RCTs) in the 5–11 age group suggests “that a range of activity interventions, and interventions that combine diet with activity, can have a modest beneficial effect on developing obesity” [46]. Very little research has taken into consideration the cost/benefit ratio of these results even if, as shown by the work of Guarino et al. [47] the economic analysis would be positive in a high percentage of them. However, there is a great heterogeneity of data and methodological settings that make a correct comparison difficult. There is a need to use global approaches (school, family, environment, society, etc.) to agree on measurable outcomes [48] and to obtain longitudinal data. It would also be useful to distinguish between true prevention of obesity (subjects therefore initially not overweight and/or obese) and prevention of the worsening of obesity.

## Genetics. 1-  Trisomy 3q syndrome

### Trisomy 3q syndrome

Serra et al. [49] report on a female preterm newborn with a de novo 3q27.1-q29 duplication. The article provides interesting insights related to the clinical and diagnostic management of the newborn carrying a genetic pathology and related individual and multidimensional follow-up strategies. In the article the analysis of correlations between genes involved in duplication and phenotypic manifestations is discussed, with a comparative review of previous described patients. The presence of risk factors related to advanced parental age, responsible for potentials chromosomal and/or genomic anomalies and assisted reproduction techniques (ART) for epigenomic defects is emphasized [50, 51]. The whole diagnostic pathway allowing the diagnosis of the contiguous gene syndrome (non-invasive prenatal diagnosis, karyotype and a-CGH) is well outlined [52]. In the clinical approach 3q27.1-q29 duplication should be included in the differential diagnosis of hypergrowth syndromes.

## Global health. 1-  Telemedicine for pediatric care

### Telemedicine for pediatric care

The use of telemedicine for pediatric care is increasing worldwide. According to the recent guidelines issued in 2020 by the Italian Ministry of Health, has been recognized as an integral part of the services of the National Health Service. The adoption of telemedicine in the field of care has found a significant impact during the covid pandemic and has allowed to continue and implement virtuous clinical care processes with improvement of the quality of health care, increasing the usability of treatments, diagnostic services and remote medical advice, along with positive economic impact too. Zuccotti et al. [53] report the peculiarities of a regional operational center for telemedicine to ensure continuing care in pediatrics. The services included routine pediatric hospital activities and innovative programs, such as early discharge, telecardiology, online supervised exercise training and preventive healthcare [54–56]. The proposed platform of telemedicine can be a useful model for other experiences in this field.

## Hematology. 1-  Thiol disulfide balance and vitamin B12 deficiency

### Thiol disulfide balance and vitamin B12 deficiency

Several studies support a relation between an increased use of cell phones and technological devices with high specific absorption rate (SAR) values, fast-food consumption, smoking cigarettes and other tobacco products and the increase in the oxidative stress levels (OSL). An increase in OSL has been linked to negative functional consequences on the central and peripheral nervous system [57–59]. Demirtas et al. [60] conducted a case-controlled observational study, on adolescents with symptoms attributable to headache by evaluating the levels of oxidation markers and B12 levels that were lower in affected ones. The statistically significant results showed that in the group with vitamin B12 deficiency native thiol levels were lower, while the disulfide and HCY levels were higher. Interestingly identification of B12 deficiency did not correlate, as in previous studies, with significant differences in MCV, or identifiable macrocytic anemia. Thus, central nervous system findings can be seen prominently in children with vitamin B12 deficiency who have normal hematological findings.

## Infectious diseases. 1- Bronchiolitis; 2-  Long COVID-19/post-COVID condition; 3-  Aggregatibacter actinomycetemcomitans infection; 4-  Antibiotic prescription and antimicrobial resistance during COVID-19 pandemic; 5-  Acute otitis media and facial nerve palsy; 6-  Metagenomic next-generation sequencing for the detection of pathogens

### Bronchiolitis

Bronchiolitis is one of the most warning cause of hospitalization for infants less than two years of age [61–63]. Seasonally, bronchiolitis hospitalization mainly correlates to RSV and mostly affects young infants less than 3 months of age not eligible to current available prophylaxis with palivizumab [63–66]. During CoronaVIrus Disease – 2019 (COVID-19) pandemic, a significant decrease of respiratory infections had been globally reported, including bronchiolitis [67, 68]. In line with respiratory tract infections decrease, there was also a significant decrease in antibiotic prescriptions, which are too often inappropriately prescribed in children due to a lack of readily available tests or to limit parental anxiety [69–72]. Later, in the following season, an anticipated peak with an increase of the overall number of cases had been described by epidemiological reports [65, 66, 68, 73]. They confirmed that most cases of bronchiolitis are caused by RSV and more frequently affect infants less than three months of age. According to the “UPDATE − 2022 Italian guidelines on the management of bronchiolitis in infants”, the diagnosis is made by anamnestic and clinical evaluation and the management is supportive [61, 74]. Since specific etiological treatment is not available, the authors suggest fluid and/or respiratory supplement, avoiding salbutamol, glucocorticosteroids and antibiotics [62, 74, 75]. Oxygen therapy should be provided in case of respiratory distress and hypoxemia and may be discontinued when saturation levels equal to or greater than 93–94% [61, 66, 74]. Recent epidemiological reports highlight that oxygen support as well as sub intensive or even intensive care hospitalization were more frequently required compared to previous seasons.

### Long COVID-19/post-COVID condition

In the COVID-19 era, we encounter a new disease, named “Long COVID” which may affect even children [76, 77]. To meet the criteria for the diagnosis, young people with a history of confirmed Acute Respiratory Syndrome Coronavirus 2 (SARS-CoV-2 infection), should present with at least one persisting physical symptom for a minimum duration of 12 weeks in the absence of an alternative diagnosis [76, 77]. Symptoms may vary, including fatigue, hemicrania, dizziness or disequilibrium, asthenia or weakness, chest pain, cough and respiratory distress under exertion [77–79].

### Aggregatibacter actinomycetemcomitans infection

Aggregatibacter actinomycetemcomitans is an oral flora colonizing bacterium which may cause dental caries and periodontitis [80, 81]. In literature it has been associated to severe extra-oral infections including endocarditis, soft tissue abscesses and more rarely to osteomyelitis, brain abscess and pneumonia [80, 82]. A prolong-term antibiotic treatment is suggested to get a complete eradication. Nevertheless, the optimal duration of therapy is not known depends on multiple variables including patient clinical response, the extent of tissue involvement [80–83].

### Acute otitis media and facial nerve palsy

Acute mastoiditis is the most frequent complication of acute otitis media while meningitis, subperiosteal, brain abscesses and facial nerve paralysis are more severe but rarely reported during childhood [84, 85]. After the widespread use of antibiotics, the prognosis of acute otitis media complications is generally good after the appropriate therapy, even though residual dysfunction may happen [84, 86–88].

### Metagenomic next-generation sequencing for the detection of pathogens

Recently, Metagenomic Next-generation sequencing (mNGS) has started to been used in the detection of bacteria to clarify the aetiology and guide anti-infection treatment [89–92]. The benefits are a rapid and accurate identification of pathogens searching for pathogens not commonly identifiable using conventional technology [89, 91, 92]. Evidence suggests that in most cases treatment may be changed on mNGS results, with a faster clinical improvement [89, 90].

## Neonatology. 1-  Vitamin D level and neonatal respiratory distress syndrome; 2-  Neurodevelopmental outcomes of very low birth weight preterms

### Vitamin D level and neonatal respiratory distress syndrome

The results of studies on the association between vitamin D levels and respiratory distress are not consistent, with differences in both maternal and fetal several variables involved, and cord blood 25(OH)D3 levels considered normal for the gestational ages [93–95]. Liu W et al. [96] address the potential relationship between cord blood 25(OH)D3 levels and the onset of neonatal respiratory distress syndrome (NRDS). This retrospective study was conducted on infants (gestational age 28–36 weeks) diagnosed with NRDS and non-NRDS preterm infants as the control group. The results of a monofactor analysis showed a correlation between lower cord blood 25(OH)D3 levels and NRDS. In addition, a multivariate logistic regression analysis identified as independent risk factors for NRDS the following: 25(OH)D3 cord blood levels < 57.69 nmol/L (24 ng/ml), gestational age < 31 weeks, birth weight < 1.86 kg, Apgar score (1 min) < 7 and Apgar score (5 min) < 8. The Authors conclude that 25(OH)D3 level is an independent risk factor for NRDS in preterm infants.

### Neurodevelopmental outcomes of very low birth weight preterms

Battajon et al. [97] conducted a single tertiary center prospective cohort study enrolling all infants < 30 weeks GA and birthweight < 1500 g admitted to NICU over a period of three years. The preterm baby is at risk of presenting neurodevelopmental disorders whose early identification allows targeted treatments. The study adopted up-to-date child development evaluation tools, and a valid methodology of statistical analysis, providing a valid model for further research in this field. The 2- and 4-year development evaluations showed a different expression in terms of percentages of subjects with developmental abnormalities, related risk factors and areas of development involved. At two years Bayley motor scale resulted worse in the lowest GA groups (*p* = 0.0282). No disability was present in 59.6%, a minor one in 31.1% and a major disability in 9.3%. Risk factors associated with disability were early neonatal sepsis (*p* = 0.0377), grade ≥ 3 intra ventricular hemorrhage (*p* = 0.0245), BPD (*p* = 0.0130), ROP (*p* = 0.0342), late neonatal sepsis (*p* = 0.0180), and length of hospitalization (*p* < 0.0001). Assessment at four-years, using WPPSI scale and scores with mABC 2, showed major disability in 19.7%, a minor one in 47.2%, or no disability in 33.1%. Disability was only associated with BPD (*p* = 0.0441) and length of hospitalization (*p* = 0.0077. A progressively worse performance was noted in relation to reduction of the GA, while using multivariate analysis, only the length of stay was predictive. At both ages there was no difference in the incidence of disabilities considering AGA and SGA groups, (*p* = 0.2689). The analysis of the conjoint distribution of disability at age of two and four years revealed how children without disabilities at the age of two (62.1%) developed impairments at the age of four in 58.4% of cases (*p* < 0.0001), with significant correlation between processing speed and manual dexterity with Spearman’s coefficient = 0.47 (*p* < 0.0001) and between processing speed and aiming and grasping with Spear man’s coefficient = 0.27 (*p* < 0.0001). This study demonstrated a clear shift in the incidence of disabilities since about half of children completely free from disability at two years of age, showed a disability related to fine motor skills that impacted an alteration in processing speed at four years. The authors suggest that attentional capacity may not be the primary cognitive problem, but a motor impairment and a difficulty with oculo-motor coordination. Children with oculo-motor impairment have less cognitive results and this does not reflect their true cognitive abilities. Therefore, for proper assessment of school learning problems, it is necessary to conduct a careful follow-up on all cognitive, motor and behavioral aspects as early as possible to detect the real problem. This allows intervention with appropriate neuropsychological techniques and thus improves school performance [97–100].

## Neurology. 1-  Psycho-emotional distress in relation to COVID-19 confinement; 2-  Children with autism spectrum disorder and their care-givers; 3- ADHD in children and adolescents;

### Psycho-emotional distress in relation to COVID-19 confinement

Since its appearance in Wuhan in mid-December 2019, COVID-19 has spread dramatically worldwide [101]. The pandemic forced the population to face unprecedented changes such as social isolation, closure of schools and public areas, and significantly impacted the well-being of children and adolescents. Compared with adults, children with COVID-19 usually had a milder or moderate course of the disease, but children were more susceptible to psychological effects than adults, suggesting that the pediatric population is more vulnerable toward mental health problems [102].

García-Rodríguez et al. conducted a systematic review to assess the impact of the lockdown measures associated with COVID-19 pandemic on children (from 2 to 12 years) and adolescent (from 13 to 18 years) [103]. Authors felt it was essential to conduct this systematic literature review since both children and adolescents belong to a fragile group in a stage of physical and mental development.

The reviewed studies focused on a population of children and adolescents evaluated during COVID-19 and the quarantine period. Main results can be summarized. Lifestyle changes and psycho-emotional manifestations: school closure and social isolation, which increased the use of screens and technologies making children and adolescent less capable of social skills and socialization. Psycho-emotional manifestations according to age differentiation: in the adolescent population higher levels of stress, depression and anxiety were found, while among children the most common symptoms were irritability, arguments with the rest of the family or rebellious behavior. Effects of confinement from a cross-cultural approach: focusing on young people from three different countries (Spain, Italy and Portugal), authors observed that Italian children had the lowest levels of anxiety and less nutritional, cognitive and sleep disorders than Spanish or Portuguese peers. Children from Portugal and from Spain reported more mood disturbances and more behavioral disturbances, respectively. Strategies for promoting resilience: the most common and successful strategy included spending a lot of time together in a limited space, improving communication between parents and children. Mental health at pediatric age is a source of constant concern for clinicians. Improving the knowledge about the impact that pandemic has had on children will allow clinicians to identify young people who need specialized help and consequently will allow to intervene before irremediable repercussions or long-term effects occur [104].

### Children with autism spectrum disorder and their care-givers

Autism Spectrum Disorder (ASD) refers to a group of pervasive neurodevelopmental disorders that involve moderately to severely disrupted functioning in areas such as social skills and socialization, expressive and receptive communication, and repetitive or stereotyped behaviors and interests [105]. Caring for children with ASD is a stressful process that heavily depends on the abilities of caregivers. The stress associated with raising a sick or disabled child creates a burden of care, which is defined as the physical, psychological, social, or economic reactions experienced by caregivers during the caregiving process [106]. Rasoulpoor et al. [107] designed a descriptive-analytical study to determine the relationship between care burden, coping styles, and resilience among mothers of children with ASD. Authors assessed caregiving burden, coping styles, and mothers’ resilience by contacting 80 volunteered mothers of autistic children. They responded to a questionnaire consisting of 3 parts: (a) the Caregiver Burden Inventory to measure the objective and subjective burden of care; (b) the Connor-Davidson Resilience Scale to measure the ability to deal with pressure and threats; and (c) the Coping Strategies Questionnaire to study how people cope with stress, in addition to providing demographic information. Questionnaires that were completed correctly and comprehensively by 69 mothers of children with ASD were analyzed. Mothers were recruited among the parents of patients at an Autism Center who met the predefined inclusion criteria (having a child aged between 3 and 15 years with a diagnosis of autism, and the mother’s psychological and physical well-being). Data analysis revealed that the average age of the participating mothers was 38.4 ± 7 years. Of these women, 94.2% were married, and 50.7% had only one child. Additionally, 56.5% had received a university education, but only 30.4% were employed. The average age of the children was 3.3 ± 1 years. Cross-referencing the demographic information with the questionnaire results revealed a significant correlation between maternal age, number of children, maternal employment status, child’s gender, and economic status. Mothers with more than one child, lower economic status, and daughters with ASD exhibited an increased burden of care. Although the average levels of resilience and coping styles were moderate, the average burden of care of mothers participating in the study was 95.5 ± 9.1, which shows that the care load is severe. Additionally, an inverse proportional relationship was observed between caregiving burden and the resilience of mothers with children affected by ASD. Therefore, the findings of this study indicate that mothers of children with autism are burdened with an increased caregiving load and exhibit moderate adaptation capabilities in response to the stress they face, which can be physical, emotional, social, and economic in nature [108].

### ADHD in children and adolescents

Attention Deficit Hyperactivity Disorder (ADHD) is a neurodevelopmental disorder. Main symptoms of ADHD, i.e., lack of attention and concentration, disorganization, difficulty completing tasks, being forgetful, and losing things, usually occur before age 12 years and interfere with daily life activities in more than one setting (home and school, or school and after-school time). ADHD can result in abnormal social interactions, increased risky behaviors, loss of jobs, and difficulties in school performance [109]. Boys are more likely to manifest symptoms and being diagnosed as having ADHD [110]. The Diagnostic and Statistical Manual of Mental Disorders IV distinguishes among inattentive (ADHD-I), hyperactive–impulsive (ADHD-H), and combined (ADHD-C) subtypes of ADHD. The diagnosis of ADHD-C requires the presence of symptoms across the domains of inattention and hyperactivity–impulsivity. Salari et al. [111] reported a prevalence of ADHD in children 3 to 12 years-old higher than in adolescents aged 12 to 18 years (7.6% *versus* 5.6%, respectively), with more cases among males than females, while previous research pointed out a lower prevalence in young children (2–7% [112]). In this systematic review, 1167 studies have been analyzed and the prevalence of several forms of ADHD was also measured. Results show that the prevalence of ADHD-I, ADHD-H, and ADHD-C is nearly equal among children. The prevalence of ADHD was higher when using the DSM-V diagnostic criterion than when using other criteria. According to these findings, while ADHD appears less common in childhood than in adulthood, its prevalence is increasing.

## Pulmonology. 1-  Vascular rings; 2-  Bronchoscopy and severe pneumonia; 3-  Bronchopulmonary aspergillosis

### Vascular rings

Vascular rings (VR) account for < 1% of all congenital cardiac defects. Abnormalities in position and/ or branching of the aortic arch can lead to a complete or incomplete VR that encircles and compresses the trachea, the bronchi and/or the oesophagus. Over the recent years, there has been an increase in detection of VR due to the increased rate of fetal diagnosis. Right aortic arch with aberrant left subclavian artery is the most common complete VR, followed by double aortic arch (DAA). Aberrant innominate artery (AIA) compression accounts 3 to 20% of cases of incomplete VR, followed by left pulmonary artery sling. Respiratory symptoms associated with VR often occur early in life (at age 1–6 months). The severity of clinical manifestations depends on the encroachment on the trachea, bronchus or oesophagus by the abnormal vascular structures. Common symptoms vary from apnoea and cyanosis to stridor, barky cough, wheezing, shortness of breath, dysphagia for solid food. An history of chronic cough, recurrent bronchopneumonia and fatigue during physical exertion is also frequently reported. Clinical presentation can vary, but disease severity does not appear strictly related with the degree of the anatomical obstruction [113]. A higher prevalence of severe symptoms such as reflex apnoea and stridor, has been reported in young children [114].

Computed tomography (CT) with angiography is an important diagnostic tool as it allows a careful simultaneous assessment of vascular abnormalities and airway involvement. Flexible laryngotracheal-bronchoscopy performed under light sedation and spontaneous breathing allows the dynamic evaluation of the tracheobronchial tree, revealing the localization, extension and the estimation of the airway malacia severity. Spirometry is recommended in children aged over 6 years for documenting the flow-volume curve shape abnormalities. The exercise challenge test is helpful to reproduce exercise induced symptoms frequently reported by patients.

As far as treatment, the evidence of a VR is not an indication for early surgical intervention. Corcione et al. have proposed a management algorithm of patients suspected of AIA based on the evidence from literature review of 20 original articles on 2166 patients with several vascular anomalies, including 1092 patients with AIA [115]. A rapid clinical improvement in AIA children treated with aortopexy has been reported, this supporting the role of AIA- induced tracheal compression in the pathogenesis of recurrent/chronic dry cough [113]. Gardella et al. [114] studied a patient population of 28 AIA children, 16 of whom undergone surgical correction. All patients with a clinical presentation sufficiently severe to justify surgical correction showed 70% or greater of tracheal narrowing at endoscopy, and this finding was found in any of the patients in the conservative management group. Porcaro et al. [116] conducted a review based on 14 articles whose endpoint was symptom management of several VR after treatment. Overall, the reviewed studies showed a positive trend of resolution of patients’ symptoms after surgical correction. Nevertheless, the difference in percentage of symptoms resolution likely reflects discrepancy among the different cohorts in term of timing of intervention, anatomical variants of the VR, and prevalence of associated lesions. Based on the available literature findings authors proposed an algorithm including the investigations required for the diagnosis, the indications for surgical treatment and the evaluations needed for monitoring both treated and non-treated patients during the follow up period. Treatment is recommended in all symptomatic patients, particularly in those with DAA or with marked Kommerell diverticulum, in cases with anterior or posterior tracheal compression greater than 50% of the lumen, or in the presence of concomitant congenital heart disease necessitating surgical repair. Conservative treatment might be indeed reasonable in asymptomatic or mildly symptomatic cases.

### Bronchopulmonary aspergillosis

Aspergillus spp. is a mold that colonize the airways provoking a spectrum of clinical syndromes. Invasive pulmonary aspergillosis occurs in immune-compromised subjects. The “gold standard” for diagnosis of invasive pulmonary aspergillosis requires a lung biopsy [117]. It is treated with liposomal amphotericin B in children < 2 years of age and voriconazole in older patients. Voriconazole or itraconazole are used for prophylaxis in children 2–12 years old, posaconazole those > 13 years of age [117]. Allergic sinusitis and allergic bronchopulmonary aspergillosis are characterized by allergic asthma, peripheral blood eosinophilia, skin test or elevated IgE to Aspergillus fumigatus, fungus-specific IgG or precipitins. Allergic bronchopulmonary aspergillosis affects asthmatics, with poor symptom control, and/or children with cystic fibrosis. It requires a CT of the chest for bronchiectasis. A prompt diagnosis of chronic pulmonary aspergillosis is difficult but necessary since it may evolve in idiopathic pulmonary fibrosis. In patients with aspergillus-associated hypersensitivity pneumonitis, a reduced pH values in exhaled breath condensate [118] that is also observed in acute asthma [119] may be helpful in interpreting the specific inhalation challenge. Other conditions [120] include acute community-acquired aspergillus pneumonia, aspergillus bronchitis.

### Bronchoscopy and severe pneumonia

The use of fiberoptic bronchoscopy (FOB) and bronchoalveolar lavage (BAL) is increasingly prevalent in pediatric settings as an aid in diagnosing numerous pulmonary diseases and as a therapeutic tool in specific conditions, particularly those affecting the small airways [121]. The capability provided by endoscopy to identify the etiology of severe pneumonia at an early stage represents an undeniable advantage in the clinical management and prognosis of the disorder.

Wu et al. [122] analyzed 229 patients admitted with severe pneumonia to the Pediatric Intensive Care Unit (PICU) at Xinxiang Hospital, China, between November 2018 and December 2021. Patients were divided into two groups based on the necessity of invasive ventilation (invasive ventilation group and non-invasive ventilation group) and further stratified according to the timing of BAL (early BAL group: received BAL within one day of admission; late BAL group: received BAL two days after admission). For each patient, the following information was collected: demographic data, duration of symptoms prior to PICU admission, reason for PICU admission, APACHE II score (that addressed patients’ severity in the PICU), SOFA score (for evaluating the organs’ failure), length of hospitalization overall and in the PICU. Additionally, data regarding patients’ clinical presentation, laboratory tests results, especially microbiology of the BAL specimens by PCR and culture, and endoscopic score assessment were evaluated. Notably, the most frequently isolated etiological agent in the study was *Mycoplasma pneumoniae* (36.67%), followed by *Staphylococcus aureus* (26.11%), *Haemophilus pneumoniae* (23.33%), and *Streptococcus spp*. (16.67%). Viral identification was less frequent, with RSV being the most prevalent (27.22%), followed by Influenza B virus (17.22%) and Influenza A virus (4.44%). A small portion of the pneumonias were due to fungal infections, with *Candida albicans* identified in 5.56% of cases. Comparison of endoscopic scores revealed a significantly higher score, indicating greater severity, in patients who required invasive ventilation. Moreover, a shorter PICU stay was observed in patients who underwent early BAL compared to those who had BAL two or more days after ICU admission.

The study also demonstrated that patients in the invasive ventilation group had higher SOFA and APACHE II scores and a longer PICU stay. Among the patients examined, 9.61% succumbed to their illness, although no statistically significant differences in mortality rates were observed between the various groups and subgroups. Wu et al. [122] have strengthened the growing body of evidence regarding the role of FOB and BAL in diagnosing and prognostically stratifying patients with pneumopathy, both in acute forms, as seen in the study patients, and in managing pediatric patients with prolonged/recurrent disease forms, such as recurrent pneumonia [123], and refractory disease forms, where along with CT scan, it represents an indispensable tool for the modern pediatric pulmonologist [124].

## Conclusions

Relevant publications in the field of pediatrics have been provided in the first semester of the last year. Important findings have allowed to improve understanding of pathogenic mechanisms leading to disease development. Novelties on biomarkers may be assessed to link laboratory results with clinical applications. In parallel, several recommendations have shed light on the management of diseases. Finally, interesting and promising results for developing personalized interventions have been reported. We think that published papers give something new that may potentially have a significant effect in healthcare practice.

## Data Availability

Not applicable.

## Abbreviations

a-CGH: Array-Comparative Genomic Hybridization
ADHD: Attention Deficit Hyperactivity Disorder
ADHD-C: Attention Deficit Hyperactivity Disorder Combined
ADHD-H: Attention Deficit Hyperactivity Disorder Hyperactive–Impulsive
ADHD-I: Attention Deficit Hyperactivity Disorder Inattentive
AGA: Appropriate for Gestational Age
AIA: Aberrant Innominate Artery
APACHE II score: Acute Physiologic Assessment and Chronic Health Evaluation II
ART: Assisted Reproduction Techniques
ASD: Autism Spectrum Disorder
BAL: Bronchoalveolar Lavage
BPD: Bronchopulmonary Dysplasia
COVID-19: CoronaVIrus Disease – 2019
CT: Computed Tomography
DAA: Double Aortic Arch
DKA: Diabetic Ketoacidosis
DSM-V: Diagnostic and Statistical Manual of Mental Disorders, Fifth Edition
EGID: Eosinophilic Gastrointestinal Disorders
EoE: Eosinophilic Esophagitis
FOB: Fibreoptic Bronchoscopy
GA: Gestational Age
HCY: Homocysteine
i.e.: Id Est
IVIG: Intravenous Immune Globulin
KD: Kawasaki disease
mABC: Movement Assessment Battery for Children
MCV: Mean Corpuscolar Volume
mNGS: Metagenomic Next-generation sequencing
NICU: Neonatal Intensive Care Unite
NRDS: Neonatal Respiratory Distress Syndrome
OSL: Oxidative Stress Levels
PCR: Polymerase Chain Reaction
PICU: Pediatric Intensive Care Unit
PPCC: Pediatric Poison Control Center
ROP: Retinopathy of Prematurity
RSV: Respiratory Syncytial Virus
SAR: Specific Absorption Rate
SARS-CoV-2: Acute Respiratory Syndrome Coronavirus 2
SGA: Small For Gestational Age
SOFA score: Sequential Organ Failure Assessment
T1D: Type 1 Diabetes Mellitus
WPPSI: Wechsler Preschool and Primary Scale of Intelligence
VR: Vascular Rings
